# Period poverty and mental health of menstruators during COVID-19 pandemic: Lessons and implications for the future

**DOI:** 10.3389/fgwh.2023.1128169

**Published:** 2023-03-01

**Authors:** Aishwarya Rohatgi, Sambit Dash

**Affiliations:** ^1^ Independent Researcher, New Delhi, India; ^2^ Division of Biochemistry, Department of Basic Medical Sciences, Manipal Academy of Higher Education, Manipal, India

**Keywords:** period poverty, menstruators, COVID-19, mental health, menstrual hygiene

## Abstract

Menstruation is a naturally occurring phenomenon; however, millions of adolescent girls and women, as well as nonbinary persons who bleed every month, are deprived of menstruating safely and respectfully. Those belonging to marginalized sections face the brunt of lack of access to water, sanitation, and hygiene facilities; affordable menstrual supplies; and inequitable distribution of menstrual health education and are victims of period poverty. Their preexisting suffering was further exacerbated by the COVID-19 pandemic, which negatively affected the mental health of those menstruating. Adolescent girls and women in communities found it persistently challenging to access and afford menstrual products while deprived of menstrual hygiene education. These put them under immense stress and elevated financial liability, predisposing them to period poverty. Interventions addressing period poverty rely on mustering collective community voices and deploying adequate policy tools. All the efforts and solutions must provide social and financial protection nets and psychological aid through mental health support groups. It is core to drive menstrual health equity for all menstruators, irrespective of who they are, what they do, and where they live.

## Introduction

Around 1.8 billion people menstruate every month worldwide, meaning at a given point each day, 800 million women and girls menstruate, comprising 26% of the global population. Most menstruators experience their first period between ages 10 and 16, lasting up to about 50 years of age, accounting for more than 50% of their average life span (1).

Periods are a difficult time for women because it has an impact on both their physical and mental health every month. Hormonal fluctuations during the premenstrual period activate several neural mechanisms that cause physical (e.g., pain and swelling) and psychological (e.g., negative affect and mood) symptoms (2). Blood loss is accompanied by muscle stiffness, cramps, painful breasts, food cravings, mood swings, irritability, fatigue, headache, and swelling.

Menstruation continues to be a normal physiological phenomenon for all menstruators, but safety, accessibility, dignity, and equity during menstruation remain elusive. This frequently causes them to suffer from what is now known as period poverty. Women and adolescent girls are exposed to precarious situations/adverse situations when they bleed, adding to their woes of experiencing excruciating pain and discomfort on a regular basis.

For a healthy and safe period, menstruators need access to clean water, sanitation, and essential menstrual products that are affordable and safe. More than 35% of the world’s population lacks these necessities. Poor hygiene measures during menstruation can pose serious health risks, such as reproductive and urinary tract infections, thrush, and others. Menstruators require access to sanitary facilities, clean water, and affordable, safe menstrual products in order to have a healthy and secure period.

Menstrual health needs can also be unmet due to traditional gender roles, cultural taboos, and poor living situations. Young adolescents, nonbinary people, and transgender men are unable to manage their periods in a dignified or safe manner (3). Menstruation-related orthodox beliefs, coupled with a lack of menstrual health supplies, menstrual hygiene education, handwashing stations, restrooms, and waste management, deter girls and women from enrolling and attending school or maintaining their employment, jeopardizing their future educational opportunities and livelihood prospects (4).

Period poverty also includes the economic vulnerability that menstruators encounter due to the financial burden of buying menstrual pads, cups, or tampons and having to bear related costs of pain medication and underwear (5). With people who identify as gender nonbinary and people with disabilities frequently being ostracized in the society, these problems exacerbate existing vulnerabilities, pushing those who menstruate to use unhealthy coping mechanisms. A study in Kenya, for example, reported that a few schoolgirls involved themselves in transactional sex to procure menstrual products in dire situations (6).

## Period poverty and COVID-19

COVID-19 exacerbated period poverty in many ways, negatively affecting menstruators’ physical and mental health. During the COVID-19 lockdowns, supply chains broke down around the world, and many microenterprises in the private sector ceased trading, distribution, and delivery of nonessential goods, particularly in hard-to-reach areas. Retail stores were shut down during the lockdown, and restrictions on movement were mandated. This made procuring menstrual hygiene goods difficult, which turned to become a scarce resource (7).

The erratic availability of menstrual supplies forced many belonging to underprivileged communities to use unsafe materials during periods. In remote regions, families facing immense economic difficulties were not able to spend money on buying pads for the women of their families. Consequently, menstruators were compelled to resort to unclean and hazardous homemade alternatives such as rags, dry leaves, ashes, linings, cotton, and mud, which were less effective and dangerous. Using such alternatives lead to reproductive tract infections and illnesses that can be life-threatening (8). For example, in India, according to data reported by the Ministry of Health and Family Welfare, only 12% of women and girls had access to sanitary napkins. At the same time, many depend on conventional unhygienic methods during menstruation (9). For women residing in slums and crowded areas and dependent on community toilets for sanitation, the social distancing restrictions of COVID-19 made menstrual health and hygiene management an arduous task (10).

The interrupting global supply chains caused the cost of menstrual products to rise at an unprecedented rate. Most young girls who bleed are school and college adolescents with financial dependency on their parents. On average, women make up 46% of the labor force around the world, and most of them are homemakers dependent on their male counterparts for their financial needs (11).

Other menstruators, including nonbinary persons, are at the highest risk in the hierarchy of financial security. These adverse circumstances in the pandemic, while disrupting livelihoods and straining household incomes, predisposed menstruators to severe financial risk as most could not afford safe menstrual products. During the pandemic and the incumbent lockdown in India, many female migrant workers who were part of the mass exodus to their villages had almost negligible access to toilets or money for food, let alone sanitary napkins (8).

During the peak of COVID-19, critical social services and community initiatives that women and girls relied on for support were paused. Similarly, caregivers who were sick and in isolation could not provide washing and toileting assistance, specifically to dependents like people with disabilities, despite these facilities being essential to good menstrual hygiene management (12).

In a survey by Plan International concerning menstrual hygiene management, water, sanitation, and hygiene (WASH) professionals from 24 countries reported that COVID-19 had aggravated the situation for menstruators. About 73% of the respondents agreed that the pandemic restricted access to menstrual supplies due to acute shortages in production and dismantled supply chains; 68% said that access to WASH facilities that facilitate privacy for cleaning and disposal of sanitary products was impaired; 58% agreed that prices of menstrual products sky-rocketed, and about 54% felt that there almost was nil menstrual health literacy. About 51% of respondents said there was reduced availability of clean water needed during periods; around 47% felt that the environment for disposal of menstrual products was unavailable, while 24% said that there was emotional distress, stigma, shame, and harmful practices linked with menstruation (13).

With the lockdown, millions of students had to stay home as most of the schools and colleges were shut down; the proximity that adolescent girls had with their teachers, family members, and peer networks was absent, which resulted in them having unverified and limited information on their menarche and its management, especially with curriculums and programs around menstrual hygiene not being incorporated into study modules of educational institutions. This challenge heightened in those locations where young people were limited by poor internet connectivity and limited access to virtually available resources (13).

## Period poverty and mental health

Period poverty has also disempowered women, disrupting their mental health and wellbeing. The lack of means for hygienic management of menstruation can cause discomfort and psychological stress and can add to the shame and sometimes depression that women and girls experience because of menstruation-related taboos and stigma (14). Another instance of discriminatory practice during periods is from Egypt, where a study found many schoolgirls reporting that they do not bathe during their menstruation because it is considered a social taboo to come in contact with water during the menstrual cycle (15).

A study in Nepal found that many girls were forced to stay in a hut or sleep in the fields during their period even though the government had decreed this practice illegal (16). Such malpractices are rampant across the globe as well in India. They have severe psychological outcomes on women and impair their mental health as they become victims of prejudiced societal norms.

COVID-19 triggered a massive 25% rise in common mental health problems of anxiety and depression worldwide (18). During the first lockdown in France, 9.6% of 890 women aged 18–50 years had difficulty accessing period protection. About 49.4% of women experiencing period poverty had depressive symptoms, compared to 28.6% of women who had not experienced menstrual poverty, and 40% had anxious symptoms (vs. 24.1%). The links between period poverty and depression were significant. It appeared worthwhile to look beyond the initial symptoms of depression and anxiety in women and inquire about the access to menstrual health products as probable causes (18).

The pandemic saw a substantial mental health crisis, wherein the most vulnerable sections felt the brunt of lack of adequate care. Most menstruators facing period poverty reported moderate to severe depression, given their agony of grappling with periods amid an unprecedented health emergency like the pandemic. Young girls felt isolated and lonely as their avenues of seeking menstrual hygiene education, like schools, colleges, and peer groups, were restricted. The associated societal stigma deeply rooted in cultures resulted in overwhelming anxiety with suicidal tendencies and suicides reported in extreme situations of period poverty (18).

Periods amid the pandemic prevented young women from going to school and participating in sports and social activities, which affected their physical and mental health. They also found having conversations about the stigma around periods and expressing their feelings difficult, especially with their parents, leaving them with a sense of isolation. Many menstruators from lower socioeconomic classes were compelled to forego food and necessities in favor of menstrual supplies risking their physical health. This led to financial burdens as well as emotional stress and anxiety. The government and civil society’s efforts toward distributing menstrual hygiene products and ensuring menstrual equity were also denuded in the wake of COVID-19 lockdowns, and a lack of menstrual health education led to increasingly poor mental health outcomes (12).

Menstruators living in urban areas, especially working women, were also presented with significant challenges. As the burden of domestic responsibilities increased, the boundaries separating personal life and jobs diminished due to working remotely from home. They suffered from an additional strain of dealing with menses during a lockdown, probably while suffering from COVID-19, which disproportionately impacted their mental health (8).

Since the stress levels were at an all-time high during the pandemic, the menstrual cycles of a large number of women became erratic. Studies claimed that stress-related irregular menstruation cycles were typical, which is problematic since it is associated with infertility and severe mental health issues, among others. One of the research also revealed that the newly introduced COVID-19 vaccine also caused menstrual irregularities for many people, though for a short duration (18, 19).

Another study sought to investigate the mental health status and menstrual changes of hospitalized female COVID-19 patients in the isolation ward of an Indonesian national referral hospital between January and August 2021, with follow-up for 3 months after discharge. Among the 158 female patients, there was an increase in patients with a cycle length of more than 32 or 24 days, as well as significant increases in menstrual irregularity and heavy menstrual bleeding. Following COVID-19 infection, 37.3% of patients reported a change in menstrual pattern. Neurotic symptoms were reported by 32.3% of the women, psychotic symptoms by 12.7%, and posttraumatic stress disorder symptoms by 38.0%. Patients suffering from mental health issues were twice as likely to report a menstrual change. This demonstrates that menstrual changes and increased symptoms of mental health disorders during COVID-19 are linked to each other (20).

## Discussion

The pandemic has not just been a public health crisis. COVID-19 in the past 2 years has highlighted the lingering challenges associated with menstrual health. It ranged from inadequate water and sanitation facilities to a lack of menstrual health education to incompetence in addressing cultural stigma (21). It has proven to be a “syndemic” with a looming mental health crisis and ostracization of those already vulnerable, including menstruators facing period poverty. This deleterious event is a stark reminder of our frail social protection net, which crumbled under an unprecedented health emergency. Though the COVID-19 pandemic is dwindling and the world seems to return to a new normal, it has left an indelible impact on our lives. Therefore, in a world constantly reclaiming the losses due to the pandemic, it becomes imperative to deliberate on actions that address period poverty.

There needs to be a global call to build community responses that encapsulate the essence of inclusivity. This is imperative to ensure those who have faced the devastating impact of the pandemic and sit at the bottom of the social and economic pyramid are reached, and their health needs are met. Menstrual hygiene management should be a part of the government’s health response during any emergency. This includes disseminating menstrual health and hygiene information as part of more extensive health campaigns. There needs to be coordination between Health and Education departments so that protection against period poverty is built into all recovery responses. As part of a remote online learning curriculum, integrating menstrual education should be a prerogative for all educational institutions. It should also be realized that menstrual hygiene education is not only for adolescent girls; boys also can reap its benefits. Imparting adequate education to all girls and boys on menstruation at an early age at home and on school premises promotes early initiation of safe menstrual health practices and challenges the persistent stigma around a naturally occurring biological process (7).

Policy efforts at the union and federal levels should push to bring down the cost of sanitary products and improve their access, specifically among those who are vulnerable and in need of community support. Encouraging local medium and microenterprises to meet the demands of menstrual supplies and eventually decline the reliance on global supply chains can help address the issue of shortages in emergencies like the pandemic (7). Other policy instruments include distributing menstrual products free of cost or at subsidized prices for those who belong to low-income groups and in public avenues like schools and shelter homes. The provision of subsidies is also an effective mechanism to incentivize local entrepreneurs, specifically self-help groups and women cooperatives, to manufacture their menstrual hygiene products and be the torchbearer of disseminating menstrual health literacy in their communities. Social enterprises and nonprofits can play a crucial role in driving the menstrual health education movement at the grassroots (21).

To steer financial inclusion, direct conditional cash transfers can also be one of the many tools which can be administered that consumers can use to buy menstrual products. This can enable poor communities to buy affordable products and shift the orthodox perceptions that prevail on the utility and value of such menstrual supplies (21).

Tax exemptions on all sanitary products should be a mandate. Public advocacy for tax reforms for menstrual products is one lever, when deployed, can help address the affordability of sanitary products for one and all. It can lay the foundation for a far-reaching information, education and communication (IEC) campaign and raise awareness about menstrual health and hygiene around the globe. An all-inclusive response to address period poverty should be adopted with a gender equality construct prioritizing the menstrual equity policy (21).

About 1.7 billion people globally do not have access to necessary sanitation services. In countries tagged as developing nations, around 75% of people lack basic handwashing facilities at home (22). Therefore, in addressing period poverty, all the proposed interventions must tackle infrastructural impediments like better water access and address archaic societal norms, stigma, and discrimination in male-dominated household settings (7). Drawing investments in essential public health infrastructure, including water and sanitation systems, is a proven strategy to address the lack of access to menstrual products in resource-constrained settings. Proper waste management practices and WASH facilities must be provided to curtail COVID-19 spread and ensure that respectful menstruation is maintained during any emergency response and in the future, as countries focus on building back better (13, 21).

Other than being a physiological phenomenon, periods or menstruation is affected by social determinants involving the requirement of better health services, education, inclusive workplaces, the emancipation of women, and in a larger sense achieving gender equality. Therefore, there cannot be a denial that menstruation, education, public health, and poverty link together (6). Despite concurrent efforts to include menstruation in these domains, period stigma, shame, and taboos are prevalent in all households, health centers, institutions, and public spaces. Period poverty inadvertently poses physical, mental, and emotional challenges for people who menstruate. Menstrual health and hygiene management is an impetus to achieving universal health coverage. Alleviating period poverty remains a social justice and human rights issue that needs to be endorsed and promoted by the governments, civil society, and, most importantly, the citizens.

## Data Availability

The original contributions presented in the study are included in the article/Supplementary Material, further inquiries can be directed to the corresponding author.
